# Endotoxin Structures in the Psychrophiles *Psychromonas marina* and *Psychrobacter cryohalolentis* Contain Distinctive Acyl Features

**DOI:** 10.3390/md12074126

**Published:** 2014-07-09

**Authors:** Charles R. Sweet, Giancarlo M. Alpuche, Corinne A. Landis, Benjamin C. Sandman

**Affiliations:** 1 Chemistry Department, 572M Holloway Road, United States Naval Academy, Annapolis, MD 21402, USA; E-Mails: gpuche84@gmail.com (G.M.A.); clandis@hmc.psu.edu (C.A.L.); bensandman2@gmail.com (B.C.S.); 2 Belize Defense Force, Belize City P.O. Box 141, Belize; 3 Penn State College of Medicine, 500 University Drive, Hershey, PA 17033, USA; 4 US Navy SUBASE NL, Box 44, Groton, CT 06349, USA

**Keywords:** lipopolysaccharide, lipid A, lipid structure, mass spectrometry, psychrophile

## Abstract

Lipid A is the essential component of endotoxin (Gram-negative lipopolysaccharide), a potent immunostimulatory compound. As the outer surface of the outer membrane, the details of lipid A structure are crucial not only to bacterial pathogenesis but also to membrane integrity. This work characterizes the structure of lipid A in two psychrophiles, *Psychromonas marina* and *Psychrobacter cryohalolentis*, and also two mesophiles to which they are related using MALDI-TOF MS and fatty acid methyl ester (FAME) GC-MS. *P. marina* lipid A is strikingly similar to that of *Escherichia coli* in organization and total acyl size, but incorporates an unusual doubly unsaturated tetradecadienoyl acyl residue. *P. cryohalolentis* also shows structural organization similar to a closely related mesophile, *Acinetobacter baumannii*, however it has generally shorter acyl constituents and shows many acyl variants differing by single methylene (-CH_2_-) units, a characteristic it shares with the one previously reported psychrotolerant lipid A structure. This work is the first detailed structural characterization of lipid A from an obligate psychrophile and the second from a psychrotolerant species. It reveals distinctive structural features of psychrophilic lipid A in comparison to that of related mesophiles which suggest constitutive adaptations to maintain outer membrane fluidity in cold environments.

## 1. Introduction

Bacterial lipopolysaccharide (LPS) is an essential component of the cellular envelope in Gram-negative bacteria such as *Escherichia coli*. It is also a molecule of fundamental importance in medicine; LPS was first identified as endotoxin due to its activation of the mammalian innate immune system and its role in septic shock [1]. The discovery that LPS is a potent bioactive material led to its intensive characterization as a class of structurally related molecules, built around an essential disaccharide glycolipid known as lipid A [2]. Lipid A is synthesized by a set of essential enzymes (nine in *E. coli*) that are predominantly encoded by *lpx* genes. Synthesis proceeds from the common metabolic intermediate UDP-*N*-acetyl-glucosamine by acylation of this structure with two acyl units (through LpxA, LpxC, and LpxD). Condensation of two of these units to disaccharide monophosphate with four acyl chains (by LpxH and LpxB), and subsequent phosphorylation to form tetra-acyl lipid A *bis*-phosphate (by LpxK) is the last generally conserved step among Gram-negative bacteria. From this common intermediate, the lipid A molecule is decorated and modified in a wide variety of divergent ways in the final steps of lipid A synthesis [3]. In the fundamental structure of *E. coli* lipid A, the molecule is finished by the addition of an acyl-oxyacyl (secondary) acyl chain to each of the two acyl hydroxyl groups on the non-reducing sugar, for a total of six acyl residues in the molecule.

While *E. coli* lipid A represents the canonical and most potently immunogenic lipid A, there is significant structural variability among Gram-negative bacteria, even within a single genus or species of the γ-proteobacteria [4]. This variability has been linked to a variety of biological functions, including virulence, interspecies interactions, and survival under hostile conditions [3,5,6,7,8,9]. For example, in *E. coli*, hostile environments can trigger metabolic modulation of the lipid A structure, including “hiding” the phosphates with positively charged ethanolamine and amino-arabinose modifications [10] and addition of a seventh acyl chain [11]. In bacteria that are *not* constitutively adapted to cold (mesophiles such as *E. coli* and *B. subtilis*), cold shock induces homeoviscous adaptation of phospholipid acyl structure in order to preserve membrane integrity; this response increases incorporation of unsaturated, short, and/or branched fatty acids to maintain membrane fluidity as part of a set of physiological actions necessary to survival in cold conditions [12,13,14,15]. Lipid A has also been shown to be altered by homeoviscous metabolic alteration in mesophiles; in *E. coli* a *cis*-unsaturated hexadecenoyl residue is added in place of the typical 2′-acyl-oxyacyl dodecanoyl residue during cold shock [16], while growth at cooler temperatures induces *Franciscella* species to use 3-OH hexadecanoyl residues in preference to 3-OH octadecanoyl ones at the 2 and 2′ positions of lipid A [7].

Despite a thorough biochemical understanding of lipid A in the Enterobacteriaceae, little is known about its detailed structure in psychrophilic species. We hypothesize that the structure of psychrophilic lipid A is similar to that of closely related mesophiles, but with constitutive differences that mirror those of the metabolic homeoviscous/cold shock response such as the use of shorter and/or unsaturated acyl units. This hypothesis was investigated by the isolation of lipid A from the obligate psychrophile *Psychromonas marina* and the psychrotolerant species *Psychrobacter cryohalolentis*, structural characterization by matrix-assisted laser desorption/ionization time-of-flight mass spectrometry (MALDI-TOF MS) and fatty acid methyl ester gas chromatography-mass spectrometry (FAME GC-MS), and comparison of these structures to those of the related mesophiles *E. coli* and *Acinetobacter baumannii*, respectively. These two psychrophiles are taxonomically divergent, though both were originally cultured from cold marine environments (*P. marina* from −1 °C sea water and *P. cryohalolentis* from a saline lens (cryopeg) embedded in a marine region of Siberian permafrost) and both are capable of growth at temperatures below 0 °C [17,18]. *P. cryohalolentis* and the mesophilic opportunistic pathogen *A. baumannii* are both Moraxellaceae [18], a family which belongs to a poly-order clade that is functionally distinct from other γ-proteobacteria in their regulation of rRNA [19], while *P. marina* is an obligate psychrophile considered to be a member of the order Alteromonadales, a predominantly psychrophilic order [17]. However, a recent γ-proteobacterial phylogeny based on 356 proteins [19] shows that Alteromonadales is polyphyletic, and the Psychromonadaceae are members of a clade that includes the orders Vibrionales, Pasteurellales, and Enterobacteriales and excludes even the closest classical family of the Alteromonadales, the Shewanellaceae. *E. coli*, the enterobacterial type species, is therefore an appropriate mesophilic comparison for *P. marina*.

## 2. Results

### 2.1. MALDI-TOF MS of E. coli Lipid A

**Figure 1:**
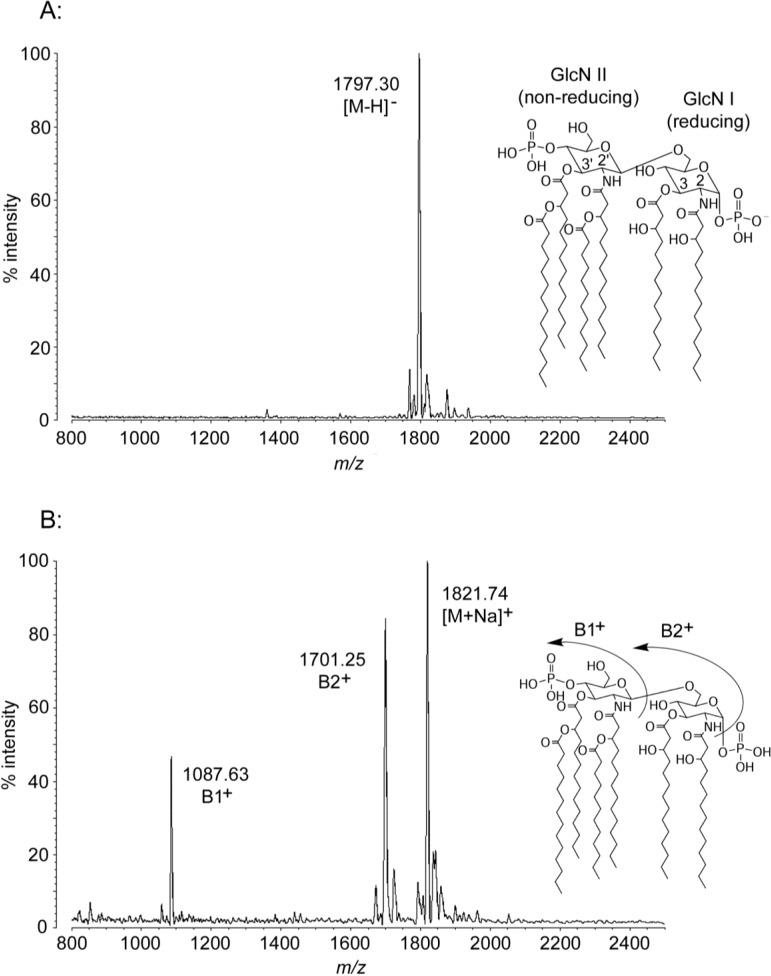
MALDI-TOF mass spectra of *E. coli* lipid A. Purified lipid A was prepared by mixing with ATT matrix as described in Materials and Methods. Spectral data were collected by Shimadzu Axima Confidence MS with a power setting of 65 and pulsed extraction at 2000 Da as the average of 581 profiles in the linear negative mode (**A**) or 1143 profiles in the linear positive mode (**B**). Peak interpretation is shown on the spectra, and in Table 1 with comparison to predicted molecular weights. To aid structural interpretation, the inset to (A) includes a summary of critical nomenclature and that of (B) shows the positive ion carbohydrate fragmentation pattern typical of lipid A molecules.

We characterized *E. coli* lipid A from the lab strain W3110 as a MALDI-TOF calibration standard and an experimental control validating our structural analysis of the MALDI-TOF and FAME GC-MS data. Negative ion MALDI-TOF MS of the purified *bis*-phosphate fraction (Figure 1A) demonstrates a mass/charge ratio (*m*/*z*) of 1797.30, consistent with previous characterizations and corresponding to the typical *E. coli* “[M − H]^−^” deprotonated molecular mass of 1797.36 Da (Figure 1A, inset). Positive ion MALDI-TOF MS of *E. coli* lipid A (Figure 1B) displays characteristic fragmentation of the labile glycosidic linkages of lipid A into B1^+^ ion (non-reducing sugar and decorations only, observed *m*/*z* of 1087.63 in *E. coli*) or B2^+^ ion (loss of the reducing-sugar phosphate, observed *m*/*z* of 1701.25), as well as a cationic adduct [M + Na]^+^ (molecular mass + sodium, observed *m*/*z* of 1821.74). A graphic summary of the diagnostic fragmentation pattern typical of positive ion MALDI-TOF MS of lipid A molecules is provided in the inset to Figure 1B. In addition to the dominant [M − H]^−^ peak, small flanking peaks +28 Da and −28 Da from the main form indicate the presence of alternative acyl chains used at low frequency by one or more acyltransferases, as has been described previously [20].

**Figure 2:**
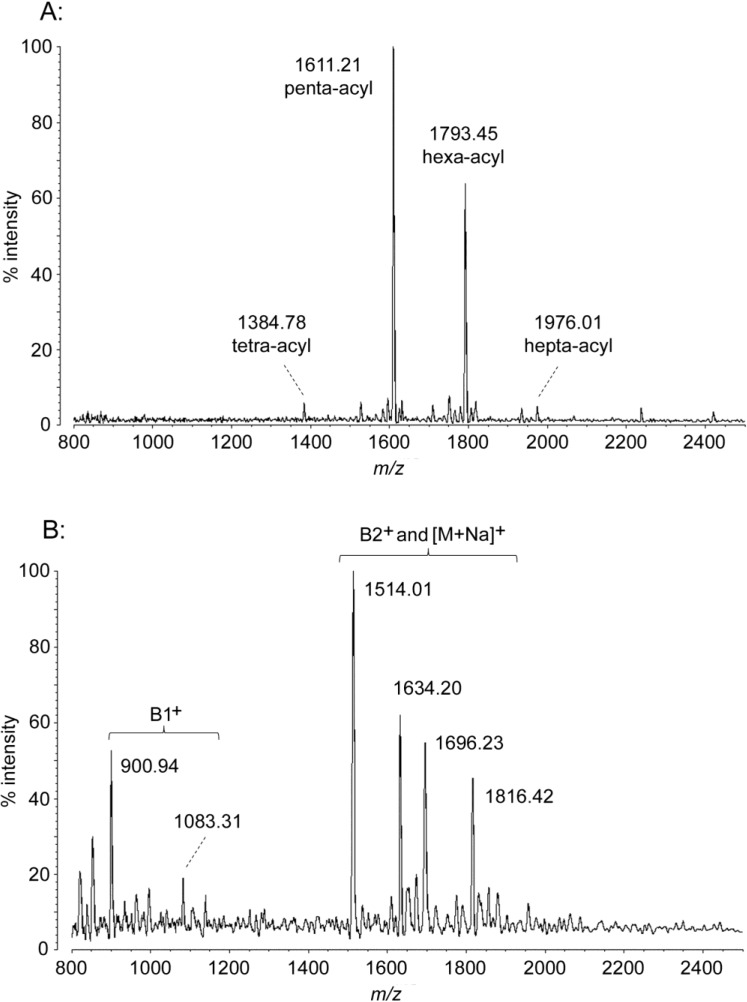
MALDI-TOF mass spectra of *P. marina* lipid A. Purified lipid A was prepared by mixing with ATT matrix as described in Materials and Methods. Spectral data were collected by Shimadzu Axima Confidence MS with a power setting of 65 and pulsed extraction at 2000 Da as the average of 1003 profiles in the linear negative mode (**A**) or 2000 profiles in the linear positive mode (**B**). Peak interpretation is shown on the spectra, and in Table 1.

**Figure 3:**
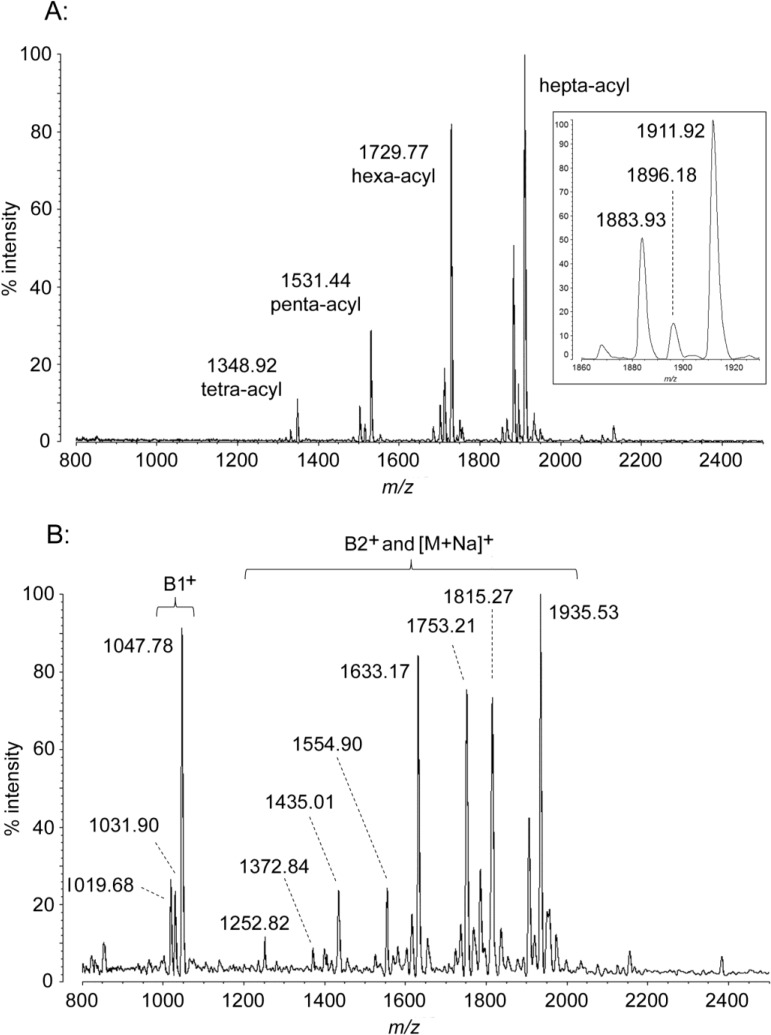
MALDI-TOF mass spectra of *A. baumannii* lipid A. Purified lipid A was prepared by mixing with ATT matrix as described in Materials and Methods. Spectral data were collected by Shimadzu Axima Confidence MS with a power setting of 70 and pulsed extraction at 2000 Da as the average of 1249 profiles in the linear negative mode (**A**) or 1583 profiles in the linear positive mode (**B**). Peak interpretation is shown on the spectra and in Table 1.

**Figure 4:**
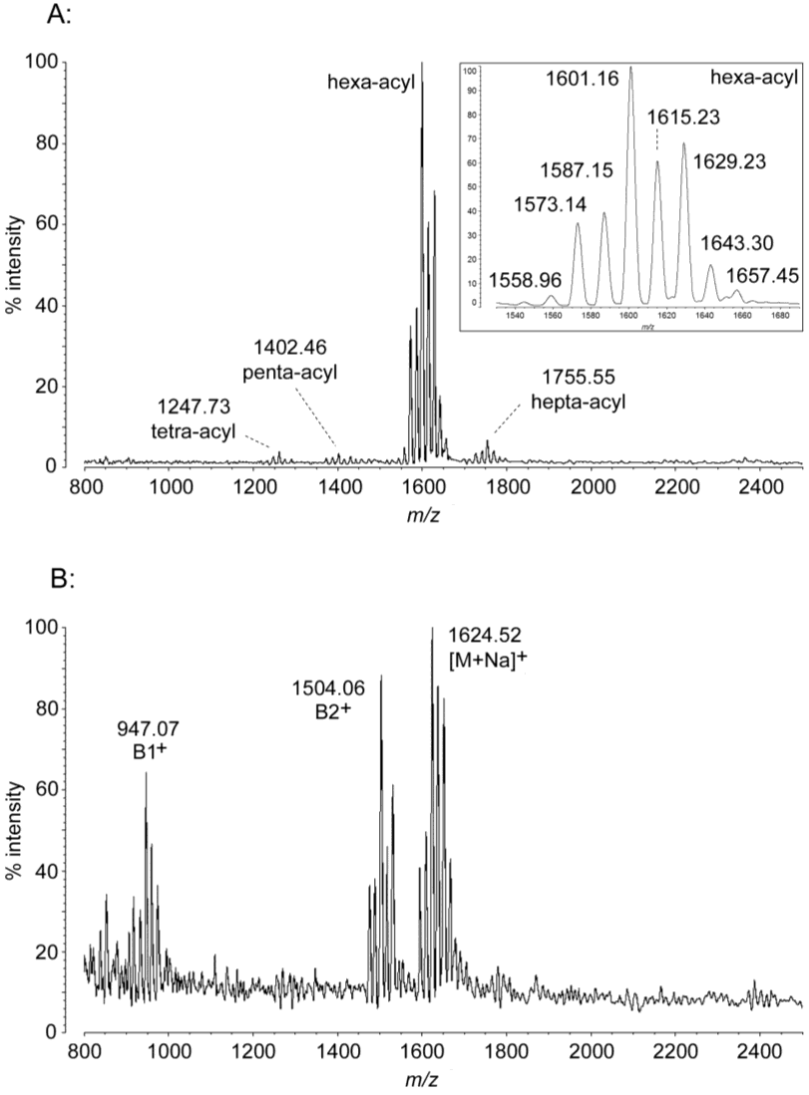
MALDI-TOF mass spectra of *P. cryohalolentis* lipid A. Purified lipid A was prepared by mixing with ATT matrix as described in Materials and Methods. Spectral data were collected by Shimadzu Axima Confidence MS with a power setting of 65 and pulsed extraction at 2000 Da as the average of 2000 profiles in the linear negative mode (**A**) or in the linear positive mode (**B**). Peak interpretation is shown on the spectra and in Table 1.

Under standard conditions, roughly one third of *E. coli* lipid A carries a third phosphate residue, and this *tris*-phosphate structure (molecular weight of 1878.34 Da) can be isolated by the methods described here in high-salt (480 mM) DE52 column fractions, however the *tris*-phosphate is only present as a minor [M − H]^−^ peak in the “*bis*-phosphate” 240 mM fraction shown in Figure 1A (observed *m*/*z* of 1877.20). Only *bis*-phosphate molecules were observed in significant amounts in any fractions of the *P. marina*, *P. cryohalolentis*, or *A. baumannii* lipid A isolates (Figure 2, Figure 3 and Figure 4 and data not shown); *E. coli bis*-phosphate was therefore used as the primary structural control in this work. Table 1 lists the major observed MALDI-TOF ion masses for all four species (further described in Section 2.2, Section 2.3 and Section 2.4), and compares them to the theoretical ion masses predicted by the structural conclusions. The spectra shown in all figures represent typical results of multiple experiments.

**Table 1:** Summary of MALDI-TOF MS observations and predicted masses of ions and fragments.

Molecule	[M − H]^−^ Predicted Mass (Da)	[M − H]^−^ Observed *m*/*z*	B1^+^ Predicted Mass (Da)	B1^+^ Observed *m*/*z*	B2^+^ Predicted Mass (Da)	B2^+^ Observed *m/z*	[M + Na]^+^ Predicted Mass (Da)	[M + Na]^+^ Observed *m/z*
***E. coli*** hexa-acyl	1797.36	**1797.30**	1087.51	**1087.63**	1701.38	**1701.25**	1821.35	**1821.74**
***P. marina*** penta-acyl	1611.02	**1611.21**	901.21	**900.94**	1515.04	**1514.01**	1635.02	**1634.20**
hexa-acyl	1793.33	**1793.45**	1083.48	**1083.31**	1697.35	**1696.23**	1817.32	**1816.42**
***A. baumannii*** hexa-acyl	1730.20	**1729.77**	1047.40	**1047.78**	1633.22	**1633.17**	1753.19	**1753.21**
hexa-acyl − (OH)	1714.21	**1713.80**	1031.41	**1031.90**	1617.22	**1616.93**	1737.20	**1737.31**
hexa-acyl − (CH_2_)_2_	1702.15	**1701.71**	1019.35	**1019.68**	1605.16	**1604.72**	1725.14	**1724.97**
hepta-acyl	1911.50	**1911.92**	1047.40	**1047.78**	1815.52	**1815.27**	1935.50	**1935.53**
hepta-acyl − (OH)	1895.50	**1896.18**	1031.41	**1031.90**	1799.52	**--**	1919.50	**1920.34**
hepta-acyl − (CH_2_)_2_	1883.45	**1883.93**	1019.35	**1019.68**	1787.47	**1787.30**	1907.44	**1907.36**
***P. cryohalolentis*** − (CH_2_)_3_	1558.91	**1558.96**	905.17	**907.11**	1462.93	**1461.39**	1582.90	**1581.95**
−(CH_2_)_2_	1572.93	**1573.14**	919.19	**919.09**	1476.95	**1476.26**	1596.93	**1596.22**
−CH_2_	1586.96	**1587.15**	933.22	**934.13**	1490.98	**1489.93**	1610.96	**1610.55**
hexa-acyl	1600.98	**1601.16**	947.25	**947.07**	1505.01	**1504.06**	1624.98	**1624.52**
+CH_2_	1615.01	**1615.23**	961.27	**961.36**	1519.03	**1517.92**	1639.01	**1638.86**
+(CH_2_)_2_	1629.04	**1629.23**	975.30	**975.01**	1533.06	**1532.13**	1653.04	**1652.71**
+(CH_2_)_3_	1643.06	**1643.30**	989.33	**990.63**	1547.09	**1546.00**	1667.06	**1667.18**
+(CH_2_)_4_	1657.09	**1657.13**	1003.35	**1003.68**	1561.11	**--**	1681.09	**1680.35**

Calibrated mass/charge ratios (observed mass) of major ions in the MALDI-TOF MS spectra (Figure 1, Figure 2, Figure 3 and Figure 4) are compared to the calculated molecular weight (predicted mass) of each ion based on the proposed lipid A structures. See Figure 6 for proposed structures and Figure 1 for structural definitions. [--] = not detected.

### 2.2. MALDI-TOF MS of P. marina Lipid A

In *P. marina*, two dominant peaks are observed by negative ion MALDI-TOF MS (Figure 2A, Table 1), a penta-acyl structure at an *m*/*z* of 1611.21 and a hexa-acyl one at 1793.45. Both of these are flanked by minor peaks of approximately +14 or +28 Da and −14, −28, or −42 Da, representing low levels of variation in acyl chain length similar to that seen in *E. coli* (Figure 1). The positive-mode spectrum shows B2^+^ fragment (loss of anomeric phosphate) and [M + Na]^+^ adduct ions corresponding to the major negative ions described above, as well as two B1^+^ ions (loss of reducing end of molecule) corresponding to tetra-acyl (*m*/*z* 1083.31) and tri-acyl (*m*/*z* 900.94) fragments of the hexa- and penta-acyl structures, respectively. (Figure 2B, Table 1). These positive ion fragments show that the secondary (acyl-oxyacyl) chains of *P. marina* are located at the non-reducing (distal) end of the molecule in penta-acyl and hexa-acyl forms. The most straightforward interpretation of these masses is that *P. marina* lipid A, like that of *E. coli*, contains 3-OH tetradecanoyl primary chains on the 2 and 3 positions of both sugars. This interpretation is also supported by FAME GC-MS data, described in Section 2.5.

Analysis of the secondary (acyl-oxyacyl) residues in *P. marina* lipid A revealed the presence of an unusual tetradecadienoyl (C14:2) acyl residue. Identification of this secondary acyl chain began in the observation that all MALDI-TOF [M − H]^−^ and fragment masses of *P. marina* show a four mass unit deficit compared to those predicted by saturated structural analogs. This is most easily visualized by comparing hexa-acyl *E. coli* at *m*/*z* 1797.30 with hexa-acyl *P. marina* at *m*/*z* 1793.45. Though the *E. coli* and *P. marina* MALDI-TOF peaks interfere with one another when run together in the same sample, the absolute four-dalton difference in mass between the two species was confirmed directly by use of *A. baumannii* lipid A as a bracketed set of internal controls run with each (data not shown). This mass difference is consistently present in all peaks of all *P. marina* spectra; even the smallest *P. marina* MS fragment, the tri-acyl B1^+^ ion, has an *m*/*z* of 900.94 when the predicted mass of a fully saturated tri-acyl B1^+^ is 905.21 Da. From this data, it is clear that one or more of the three acyl units that remain on the triacyl B1^+^ fragment of *P. marina* lipid A must bear unsaturations, and interpretation of other positive ions shows that these unsaturations are both on the secondary acyl-oxyacyl chain. If one or more of these double bonds were in the primary residues, the B2^+^, [M + Na]^+^, and [M − H]^−^ ions would be 2 Da (one primary double bond) or 4 Da (two primary double bonds) lighter than they are due to the primary acyl symmetry of lipid A biosynthetic assembly from diacyl-UDP-glucosamine precursors. While various mechanisms of generating asymmetric primary acyl unsaturation could exist, none have been reported; the presence of a single polyunsaturated secondary acyl residue, while unprecedented in lipid A, is the most reasonable interpretation of these MALDI-TOF data. Furthermore, the presence of a tetradecadienoyl residue is supported by analysis of the FAME GC-MS data (see Section 2.5). In addition to the unsaturated secondary acyl chain, *P. marina* lipid A bears as many as two dodecanoyl acyl-oxyacyl residues. The 182 Da difference in mass between the penta-acyl and hexa-acyl [M − H]^−^ ions observed in the negative mode establishes the first of these, and the second is demonstrated by the presence of a minor hepta-acyl version of the structure at a *m*/*z* of 1976.01, 182 Da larger than the hexa-acyl ion. Though this hepta-acyl form is only a minor component of the 240 mM DE52 fraction characterized in Figure 2, it is a significant amount of the total lipid A observed in unpurified extracts (data not shown).

Another structurally significant minor form is observed in the negative-mode spectrum at *m*/*z* 1384.78. This peak represents enzymatic loss of a primary 3-OH myristoyl residue from the penta-acyl structure to yield a lipid A with three primary and one secondary acyl chains. The known lipid A deacylases, lpxR and PagL, are O-deacylases which hydrolyze the ester linkage at the 3 or 3′ position [21,22], meaning this acyl unit was removed from the 3′ position of the structure since the positive ion data localizes the secondary acyl chains to the non-reducing sugar. In combination with the positive ion masses and symmetry of lipid A biosynthesis, these data confirm that all of the primary acyl chains are 3-OH tetradecanoyl residues and that the tetra-acyl form, though missing its 3′-*O*-acyl-oxyacyl unit, still has an acyl-oxyacyl unit in the 2′ position. Note that this places the tetradecadienoyl residue on the 2′-*N*-tetradecanoyl chain, as this fragment also shows the characteristic four Da deficit compared to saturated structural predictions. Interestingly, there is no peak present that would represent the classical tetra-acyl lipid A structure (four primary and no secondary acyl chains) which would, if it existed in *P. marina*, have a molecular weight of 1405.71 Da like that of the *E. coli* precursor lipid IV_A_. Nor is there a peak at 1400 or 1402 Da which would represent a tetra-acyl structure with primary unsaturation, an absence congruent with the hypothesis of secondary acyl unsaturation and the data presented above.

### 2.3. MALDI-TOF MS of A. baumannii Lipid A

The lipid A from *A. baumannii* demonstrates two major forms in negative ion MALDI-TOF MS, a hepta-acyl species with *m*/*z* of 1911.92 and a hexa-acyl one at 1729.77 (Figure 3A, Table 1). Minor flanking peaks of ~16 Da less (indicating heterogeneity of oxidation state) and ~28 Da less (showing variation by two methylenes of acyl chain length) are associated with each of these forms. The inset to Figure 3A shows an expansion of the largest of these sets, lipid A with [M − H]^−^ *m*/*z* of 1911.92 (hepta-acyl), 1896.18 (−16 Da), and 1883.93 (−28 Da). The mass difference between the observed hexa-acyl and hepta-acyl forms, 182 Da, demonstrates that they vary by the presence of a secondary dodecanoyl chain. Likewise, the minor clusters of negative ions at *m*/*z* of 1531.44 (penta-acyl) and 1348.92 (tetra-acyl) reveal the masses and identities of more secondary acyl units by their absence: the single acyl-oxyacyl residue in the penta-acyl form is also a dodecanoyl chain, while the difference in mass between the penta-acyl and hexa-acyl forms represents a hydroxylated dodecanoyl acyl-oxyacyl residue, shown by FAME GC-MS to be a 2-OH dodecanoyl chain as described in Section 2.5. This acyl modification known to occur in lipid A due to the action of an unusual membrane-bound dioxygenase, lpxO [23], and its presence in *A. baumannii* lipid A and localization to the 2′ acyl-oxyacyl position has been shown by the work of others [24]. This acyl oxidation is not complete, as shown by the presence of minor structural variants which contain a dodecanoyl rather than hydroxydodecanoyl residues (hexa-acyl mass of 1714.21 Da and hepta-acyl mass of 1896.51 Da). The positive-mode *A. baumannii* spectrum (Figure 3B, Table 1) shows B2^+^ and [M + Na]^+^ peaks that correspond to the significant forms described above in the negative-mode spectrum. In addition, the positive ions provide more insight into the acyl structure of the molecule. Both the hepta-acyl and hexa-acyl forms yield a single tetra-acyl B1+ fragment at 1047.78 *m*/*z*, including lesser −16 Da and −28 Da variant forms. This demonstrates that both forms carry two secondary acyl units—including the 2-OH residue—on the non-reducing end of the disaccharide. The hepta-acyl form must therefore carry the remaining (seventh) dodecanoyl secondary chain on the reducing-end sugar. There are no positive ion peaks corresponding to the minor negative-mode clusters—only the major forms are abundant enough to be observed in this mode.

### 2.4. MALDI-TOF MS of P. cryohalolentis Lipid A

Unlike the small number of significant acyl variants of lipid A in most bacteria, *P. cryohalolentis* synthesizes at least seven major forms based on a high degree of acyl flexibility. These can be seen in the major cluster of hexa-acyl lipid A ions observed in negative ion MALDI-TOF MS, centered on a peak of *m*/*z* 1601.16 (Figure 4A, Table 1). This most abundant mass corresponds to a structure containing, on average, four 12-carbon and two 10-carbon acyl units. The inset to Figure 4A shows this cluster in detail; it is composed of at least seven related forms varying by ~14 Da each, indicative of single-carbon variation in the cumulative length of the acyl residues in *P. cryohalolentis* lipid A.

In addition to the major cluster of length-variable lipid A structures discussed above, there are three minor clusters seen in the negative-mode spectrum that represent alternative acylation states of the main form. The heavier cluster (*m*/*z* of 1755.55) is hepta-acyl, with the addition of a decanoyl acyl-oxyacyl unit to the most abundant average structure described above. The lighter clusters are enzymatically deacylated versions of this main cluster, with penta-acyl lipid A (*m*/*z* of 1402.46) from the removal on average of a primary 3-OH dodecanoyl unit and tetra-acyl lipid A (*m*/*z* of 1247.73) from the removal of an acyl-oxyacyl unit. These forms are likely again to be O-deacylated products of enzymes similar to lpxR and/or PagL, as described earlier for *P. marina*. The positive-mode spectrum for *P. cryohalolentis* shows the same cluster pattern as in the negative mode, with typical B1^+^ (tetra-acyl) fragments of the main [M − H]^−^ cluster centered on *m*/*z* 947.07, B2^+^ fragments centered on *m*/*z* 1504.06, and a [M + Na]^+^ adduct cluster centered on *m*/*z* 1624.52 (Figure 4B, Table 1). There are no positive ions corresponding to the minor negative-mode clusters; as with *A. baumannii* only the major forms are abundant enough to be observed in this mode.

### 2.5. FAME GC-MS Analysis of Lipid Acyl Content

To further characterize the acyl content of lipid A in these organisms, we performed GC-MS on methyl-esterified derivatives of the acyl chains (FAMEs) present in the Bligh-Dyer isolations. The transesterified fatty acid samples were characterized both by GC retention time compared to those of authentic standards and by EI-MS fragmentation pattern, which could in many cases be used to independently assign FAME identity through EI-MS library matching. Table 2 shows a summary of the FAME derivatives detected in the Bligh-Dyer extracts from each species.

**Table 2:** FAME GC-MS observations and structural interpretations.

Acyl Residue	Peak ID	Standard Retention Time	*E. coli*	*P. marina*	*A. baumannii*	*P. cryohalolentis*
**From Lipid A:**						
decanoate (C10:0)	1	3.160	--	--	--	3.147
3-hydroxyundecanoate (3-OH C11:0)	2	4.5 *	--	--	--	4.493
dodecanoate (C12:0)	3	4.264	4.265	4.273	4.264	4.260
2-hydroxydodecanoate (2-OH C12:0)	4	4.853	--	--	4.855	--
3-hydroxydodecanoate (3-OH C12:0)	5	4.990	--	--	4.990	4.989
3-hydroxytridecanoate (3-OH C13:0)	6	5.4 *	--	--	--	5.440
tetradecanoate (C14:0)	7	5.237	5.240	5.240	--	5.230
tetradecenoate (C14:1)	8	5.193	--	--	--	--
tetradecadienoate (C14:2)	9	not available	--	5.160	--	--
3-hydroxytetradecanoate (3-OH C14:0)	10	5.856	5.860	5.853	5.843	5.847
**From Phospholipids:**						
hexadecanoate (C16:0)	11	6.026	6.040	6.040	6.024	6.033
hexadecenoate (C16:1)	12	5.975	--	5.980	--	--
octadecanoate (C18:0)	13	6.733	6.733	6.733	6.715	6.720
octadecenoate (C18:1)	14	6.667	--	6.667	--	6.653

**Figure 5:**
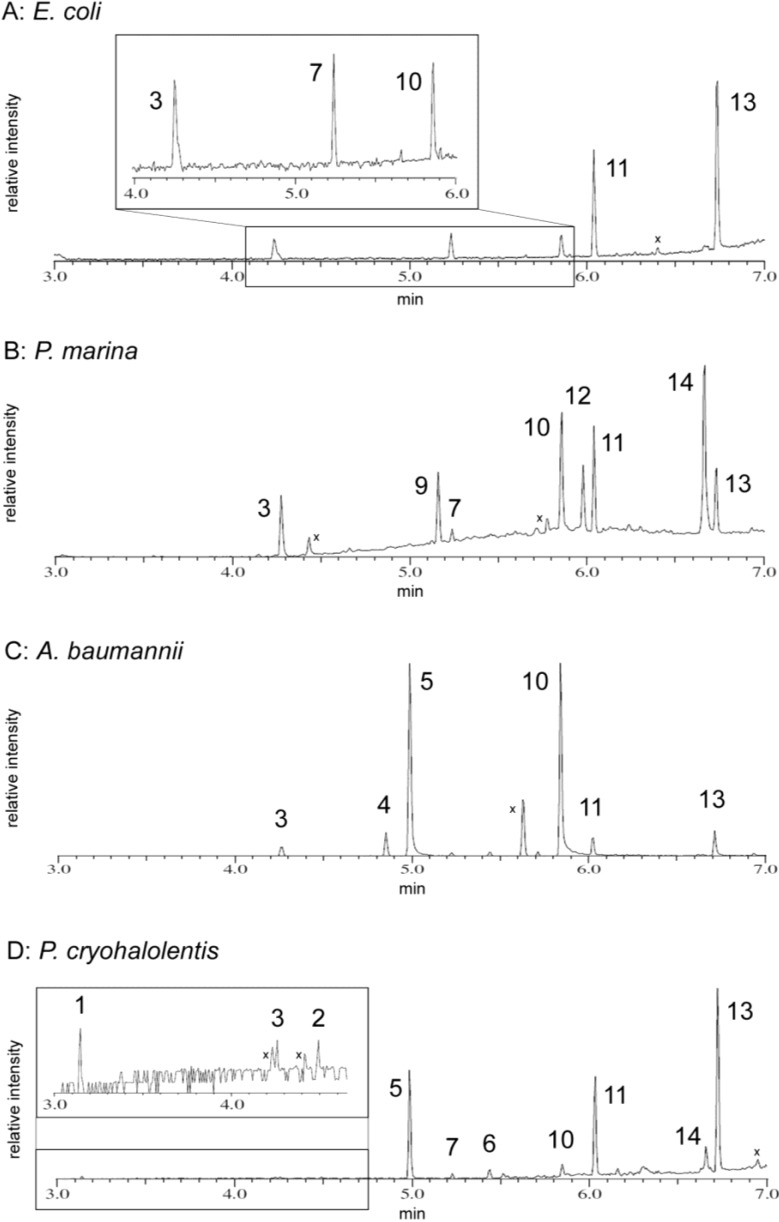
GC-MS Chromatogram of FAMEs derived from lipid extracts: Lipid extracts were converted to FAMEs and characterized by GC-MS as described in the Materials and Methods. FAMEs from *E. coli* (**A**); *A. baumannii* (**B**); *P. marina* (**C**); and *P. cryohalolentis* (**D**) extracts are indicated on the chromatogram by number, corresponding to identification in Table 2. The insets to (**A**) and (**D**) are expansions of regions with low-intensity peaks. Non-FAME peaks are marked with an “x”.

Retention times of fatty acid methyl esters in comparison to authentic standards. Peak IDs correspond to peak labels in Figure 5. Identities were determined by comparison to standards, EI-MS interpretation, and library matching (see text). * Odd-chain 3-OH standard retention times were obtained by interpolation of even chain-length hydroxy-acyl FAME retention times (data not shown). [--] = not detected.

#### 2.5.1. *E. coli* Acyl Analysis

In *E. coli*, the signature lipid A acyl units of 3-OH tetradecanoyl methyl ester (ME), tetradecanoyl ME, and dodecanoyl ME were observed (Table 2, Figure 5A). However, the *E. coli* chromatogram also contains strong peaks for hexadecanoyl and octadecanoyl residues. These longer acyl residues are not derived from lipid A but rather from phospholipids co-purified in the Bligh-Dyer extract (these phospholipids are not seen in the MALDI-TOF analysis of these samples as they are not effectively ionized with the ATT matrix).

#### 2.5.2. *P. marina* Acyl Analysis

In the *P. marina* FAME GC-MS, 3-OH tetradecanoyl ME, tetradecanoyl ME, and dodecanoyl ME are detected (Table 2, Figure 5B), along with a fourth short acyl ME that does not correspond by retention time (5.160 min) to any authentic standard. This fourth acyl residue has an EI-MS fragmentation pattern that does not match well to any standard in the MS library, but is characteristic of an unsaturated ME. The difference in retention time from this peak to tetradecanoyl ME, −0.080 min, is almost twice the difference in retention time of −0.044 min between tetradecanoyl ME and a commercial monounsaturated tetradecenoyl ME standard (Table 2). This extent of FAME retention time shift is indicative of two unsaturations, as shown by analysis of the Sigma BAME standard mix, where the gap between octadecanoyl ME and octadecadienoyl ME is −0.079 min (data not shown). The presence of this unusual acyl unit is consistent with detailed interpretation of the *P. marina* MALDI-TOF MS data described in Section 3.1, which demonstrates a four-dalton “deficit” residing in one acyl unit of the structure compared with the mass predicted by a fully saturated analog.

These data together indicate the presence of a doubly unsaturated tetradecadienoyl acyl chain in *P. marina* lipid A. Like *E. coli*, the *P. marina* GC trace also contains longer acyl residues from phospholipids, though unlike the other species in this work the phospholipid-derived FAMEs from *P. marina* are mostly unsaturated, with similar amounts of hexadecenoyl ME as hexadecanoyl ME and significantly more octadecenoyl ME than octadecanoyl ME.

#### 2.5.3. *A. baumannii* Acyl Analysis

In *A. baumannii*, the GC chromatogram shows the lipid A-derived FAMEs to be 3-OH dodecanoyl and 3-OH tetradecanoyl ME, as well as lesser amounts of dodecanoyl and 2-OH dodecanoyl ME (Table 2, Figure 5C). The absence of 3-OH tridecanoyl derivatives supports the structural conclusion published by others that the primary acyl residues are equal amounts of 3-OH dodecanoyl and 3-OH tetradecanoyl residues, and the absence of decanoyl or 3-OH decanoyl derivatives in *A. baumannii* indicates that the minor −28 Da peaks in each cluster are the result of substitution of a 3-OH dodecanoyl chain for a 3-OH tetradecanoyl one. Like *E. coli*, the *A. baumannii* FAME profile contains significant amounts of hexadecanoyl and octadecanoyl ME, again presumably from co-purified phospholipids.

#### 2.5.4. *P. cryohalolentis* Acyl Analysis

The FAME profile from *P. cryohalolentis* is consistent with the acyl chain-length heterogeneity seen in the MALDI-TOF data, and contains additional information on the details of acyl content (Table 2, Figure 5D). 3-OH dodecanoyl ME is the most abundant hydroxyacyl FAME residue, but 3-OH undecanoyl ME, 3-OH tridecanoyl ME, and 3-OH tetradecanoyl ME are present as well, suggesting the single-carbon variability in acyl chain length occurs in one or more of the primary acyl positions attached directly to the sugars. Six non-hydoxylated FAMEs were also detected in *P. cryohalolentis*. The first three, decanoyl ME (most abundant), dodecanoyl ME, and tetradecanoyl ME, contribute to acyl variation in the lipid A structure. The presence of only even chain-length non-hydroxy-FAMEs is consistent with the increased relative abundance of the +28 Da and +56 Da variants of the [M − H]^−^ cluster seen in the negative ion MALDI-TOF (Figure 4A, inset) as variation in the secondary acyl units. As with the other organisms, the FAME profile for *P. cryohalolentis* contains phospholipid acyl residues as well, with hexadecanoyl ME, octadecanoyl ME, and a small amount of octadecenoyl ME present.

### 2.6. Discussion

**Figure 6:**
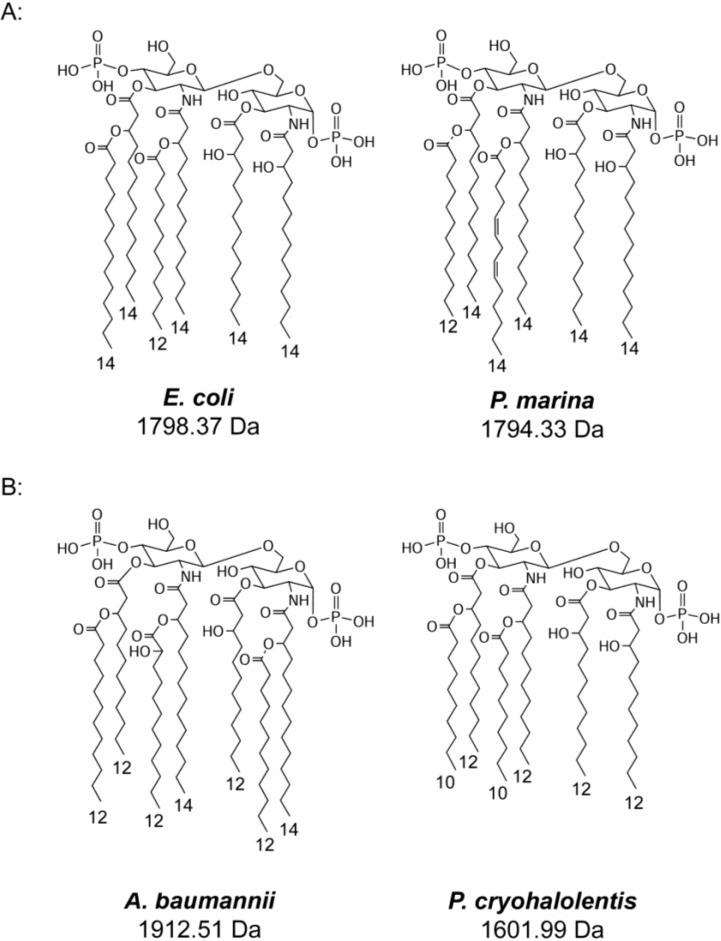
Comparison of proposed psychrophilic and mesophilic lipid A structures: These structures represent the most abundant molecular forms of lipid A in *E. coli* and *P. marina* (**A**) or *A. baumannii* and *P. cryohalolentis* (**B**) based on the summation of all structural data. Hashed bonds indicate non-stoichiometric substituents that give rise to major molecular forms.

Figure 6A presents the proposed structures of *P. marina* and *P. cryohalolentis* lipid A in comparison to those of the related mesophiles *E. coli* and *A. baumannii*. The *P. marina* structure contains 3-OH tetradecanoyl primary acyl chains and a secondary dodecanoyl acyl residue like that of *E. coli*, but differs in that the additional secondary acyl residue in the hexa-acyl structure is a tetradecadienoyl residue as opposed to the tetradecanoyl one seen in the mesophile. The presence of this unusual acyl residue is supported by multiple lines of evidence, including MALDI-TOF analysis which localizes both unsaturations in the structure to a single acyl residue, the absence of monounsaturated dodecenoyl or tetradecenoyl residues from the FAME GC-MS analysis, and an unusual FAME peak with no good match to spectral library standards, an EI-MS signature that indicates an unsaturated FAME, and a significantly faster retention time than that of the tetradecenoyl FAME standard. Analysis of minor MALDI-TOF peaks also suggests that the placement of the secondary residues in *P. marina* is different than that of *E. coli*, with the dodecanoyl chain in the 3′ acyl-oxyacyl position and the tetradecadienoyl residue at the 2′ acyl-oxyacyl one. In the absence of tetradecadienoyl standards for FAME GC-MS, no conclusions can be drawn about the structural details of this unusual chain; in Figure 6A, this acyl residue is conjectured to be *cis*, *cis*-4,7-tetradecadienoyl chain by logical analogy to the known *cis*-7-tetradecenoyl fatty acid in marine bacteria [25,26], including psychrophiles [27,28]. The most likely enzymatic means to synthesize a tetradecadienoyl derivative of common fatty acids would be through a short-chain desaturase with a tetradecenoyl substrate, perhaps analogous to the action of the eukaryotic fatty acid Δ^4^-desaturase Fad4 [29]. However, other 14:2 configurations are also biologically reasonable, including an 8,11 tetradecadienoyl residue similar to the pheromones of some insects [30]. Additional structural and enzymatic studies of this specific fatty acid and its biosynthesis will be needed to further understand this unusual structure. *P. marina* lipid A also differs from that of *E. coli* in that much of its lipid A only incorporates one acyl-oxyacyl unit (penta-acyl lipid A, shown in Figure 6A by omitting the chain attached by the hashed bond). In this penta-acyl structure, the single secondary acyl chain is the distinctive tetradecadienoyl one.

Like *P. marina*, *P. cryohalolentis* lipid A demonstrates features which are likely to increase acyl fluidity compared to that of the closely related mesophile, *A. baumannii*. However, this is the result not of unsaturation but of shorter and more varied acyl units that in aggregate make the lipid A of *P. cryohalolentis* an average of 128 Da smaller than that of *A. baumannii*. The lipid A structure of *P. cryohalolentis* is in fact quite different from those of most bacteria, with a high degree of acyl variation including odd-chain acyl variants. The dominant [M − H]^−^ peak in the negative-mode spectrum (*m*/*z* of 1601.16) is shown by the MALDI-TOF and FAME GC-MS data to be a hexa-acyl molecule carrying four 3-OH dodecanoyl (on average) residues in primary linkage and two decanoyl acyl-oxyacyl residues in secondary linkage at the non-reducing end of the molecule, a predicted molecular weight of 1601.99 Da (Figure 6B). This primary structure is flanked by at least six lesser but significant MALDI-TOF peaks that differ by a ~14 Da interval, consistent with extensive variation by methylene (-CH_2_-) units. FAME GC-MS further shows this single-methylene variation to be generated by flexibility for 11-, 12-, 13- and 14-carbon 3-OH-acyl residues in primary linkage and for 10, 12, and 14-carbon acyl residues in secondary linkage. This distinctive pattern of single-carbon acyl heterogeneity, while unusual, is not unprecedented in lipid A structure. A similar pattern is seen in the lipid A of *Pseudoalteromonas haloplanktis*, the only other psychrotolerant lipid A structure that has been reported in detail in the literature [31]; perhaps, high acyl variability is a strategy of enhancing fitness not just in the cold but across the wide −10 °C to +30 °C growth range that psychrotolerant organisms enjoy.

To accommodate synthesis of this diversity of lipid A acyl forms, *P. cryohalolentis* likely employs both multiple isoforms of one or more acyltransferases and low enzymatic substrate specificity. The *P. cryohalolentis K5* genome encodes two homologs of LpxA and only one of LpxD, LpxL, and LpxM, so isoform differences alone cannot explain the diversity of acyl forms. At least one of these enzymes must also have activity with multiple substrate chain lengths. Such lack of specificity in lipid A acyltransferases, while unusual, has been observed before in enzymatic studies of LpxA from *Acidithiobacillus ferrooxidans* [20] and *Bordetella* [32].

To compare *P. cryohalolentis* lipid A structure to that of a close relative, we also purified and characterized the lipid A structure of the opportunistic pathogen *A. baumannii*. There is only one extant structural characterization of lipid A from a clinically relevant strain of this important pathogen in the literature [24]. Factors in *A. baumannii* pathogenesis are of great biomedical interest, as some strains of this species represent a major multi-drug resistant infection threat [33], particularly in the setting of invasive medical procedures or contaminated wounds such as those sustained in combat or natural disaster [34]. Though *A. baumannii* is one of a very few Gram-negative bacteria that has been shown to survive without lipid A under some circumstances [35], endotoxin is believed to be important to pathogenesis in this organism [36], and even the absence of an endotoxin response could play a pathogenic role, as it does in other organisms [4]. In lipid A purified from *A. baumannii* we observed two major forms, hexa-acyl and hepta-acyl structures containing two 3-OH dodecanoyl and two 3-OH tetradecanoyl residues in primary linkage and secondary acylation with docdecanoyl and 2-OH dodecanoyl residues (Figure 6B). Recently published degradation experiments [24] revealed this same basic acyl composition for *A. baumannii*, and their work further concludes that the 3-OH tetradecanoyl chains are in 2 and 2′ (amide) positions while the 3-OH dodecanoyl chains are in 3 and 3′ (ester) positions. In addition to serving as a mesophilic comparison for *P. cryohalolentis*, the structure of *A. baumannii* lipid A that we have elucidated here supports and extends work recently published by others using different extraction and spectrometry methods [24].

In this work, ATCC-standard growth temperatures were chosen for all organisms in order to minimize metabolic homeoviscous adaptation of the acyl units and assess only constitutive evolutionary changes to lipid A. The possibility remains that in addition to the adaptations revealed in these experiments, psychrophiles may also display metabolic alteration of lipid A structure in response to changes in growth conditions. Analysis of possible temperature-dependent structural effects is, however, beyond the scope of this work. A previous attempt to examine homeoviscous adaptation of psychrophilic lipid A in *P. haloplanktis* was inconclusive [37], though others have met with success in examining cold-induced alteration of lipid A in mesophilic *Franciscella* species [38]. We will investigate the possibility of metabolic adaptation of psychrophilic lipid A in future experiments, using *P. marina* and *P. cryohalolentis* as models.

## 3. Materials and Methods

### 3.1. Culture Conditions, Strains, and Reagents

*E. coli* W3110 was grown at 37 °C in lysogeny broth (Fisherbrand LB-Miller, Fisher Scientific, Waltham, MA, USA) with 215 rpm rotatory shaking for 24 h as a control strain for all experiments. *P. marina* (ATCC BAA-724) was grown in Difco Marine 2216 medium at 15 °C with 215 rpm rotatory shaking for 24 h. *A. baumannii* (ATCC 19606) was grown in Difco trypticase soy broth (TSB) at 37 °C with 215 rpm rotatory shaking for 24 h. *P. cryohalolentis K5* (ATCC BAA-1226) was grown in Marine medium at 25 °C with 215 rpm rotatory shaking for 24 h. All organisms were grown at their particular standard growth temperature (as given in strain information from the American Type Culture Collection [39]) to minimize metabolic alteration of lipid A structure and assess only constitutive variations. The duration of psychrophile growth was determined from a time course study of optimal harvest density (data not shown). All media were purchased as dehydrated mixtures; culture plates were prepared by mixing media with 15% *w/v* Difco agar. Bulk solvents, reagents, and standards were purchased from Fisher Scientific or Sigma-Aldrich (St. Louis, MO, USA) and were HPLC grade or better. Bioinformatics of Lpx biosynthetic genes was assessed using NCBI’s microbial genomes databases and BLAST search algorithms [40].

### 3.2. Lipid A Isolation and DE52 Purification

Bacterial cell mass was collected by centrifugation at 2600× *g* and resuspended in PBS. This suspension was mixed with chloroform and methanol to form single-phase solution (1:2:0.8 C/M/aq) as the starting point for two-phase Bligh-Dyer extraction with mild acid release of lipid A, conducted as described previously [41]. Crude lipid A isolate was purified by DE52 anion-exchange chromatography and repurified by further Bligh-Dyer extraction, also as described previously [41].

### 3.3. MALDI-TOF MS

Purified lipid A samples were dissolved in a small amount (30–100 μL) of 2:1 chloroform/methanol. A sample of this solution was mixed *in situ* on a MALDI target slide with 20 mg/mL 6-aza-2-thiothymine in 9:9:2 water/acetonitrile/10% ammonium citrate in ratios ranging from 4:1 to 1:4, with a maximum total load volume of 2 μL. Spectral data were collected by Shimadzu Axima Confidence MALDI-TOF MS in the negative or positive linear mode (pulsed extraction 2000 Da) as the average of up to 2000 shots at moderate power (see individual figure legends for details). Shimadzu MALDI-MS software was used to perform the following processing of the raw data: (1) Calibration of all spectra to mode-specific standard datasets built from spectral data for wild-type *E. coli* hexa-acyl *bis*-phosphate lipid A (Figure 1) and *E. coli* tetra-acyl *bis*-phosphate (data not shown); (2) Averaging of all profiles collected for each sample; (3) Processing with a baseline filter width of 100 channels, threshold-apex peak detection, and an averaged peak smoothing width of 30–100 channels to resolve molecular weights from the clusters of individual isotopic masses.

### 3.4. FAME (Fatty Acid Methyl Ester) GC-MS

FAMEs were prepared by transesterification of purified lipids by incubation for 20 h with 300–500 μL 3 N HCl in anhydrous methanol and extracted from the methanol solution using an equal volume of *n*-hexane. The hexane phase was dried under nitrogen, resuspended in a small amount (~100 μL) of 2:1 chloroform/methanol and analyzed on a Shimadzu QP2010-SE gas chromatograph with a 30 m SHRXI-5ms (5% phenyl) column with electron-impact mass spectrometry (EI-MS) peak detection and characterization. Shimadzu GC-MS post-run analysis software was used to analyze the mass spectrum of each chromatogram peak and automatically matched the spectra to a library of standard MS fragmentation patterns. In some cases, signal quality was sufficient to allow library identification with strong confidence (>95%). For many peaks, however, low signal strength supported only a qualitative assessment of acyl type (saturated, unsaturated, 2-OH, or 3-OH methyl ester) from the MS spectrum alone. To further identify these peaks, retention times were compared to those of commercial fatty acid standards run on the same instrument and column, including BAME (bacterial fatty acid methyl ester) standard mix, 37-component FAME standard mix, and C8-C22 FAME standard mix (all from Sigma-Aldrich, St. Louis, MO, USA).

## 4. Conclusions

We have characterized two taxonomically distinct psychrophilic bacterial lipid A structures, those of *P. marina* and *P. cryohalolentis*. The predominant, hexa-acyl *bis*-phosphate form of *P. marina* lipid A strongly resembles that of the related mesophile *E. coli*, with the critical difference of a distinctive polyunsaturated tetradecadienoyl acyl chain. The presence of this unusual acyl chain in *P. marina* mirrors the alteration of *E. coli* lipid A during the metabolic survival response of cold-shock, in which an unsaturated hexadecenoyl acyl chain is used in place of a saturated dodecanoyl one [16]. Given that the canonical lipid A structure of *E. coli* is a potent inflammatory agonist of TLR4 [1,2] it is likely that the very similar structure of *P. marina* is inflammatory as well, though this organism is not known to be infectious.

*P. cryohalolentis* lipid A is similar in overall organization to the related mesophile *A. baumannii*, but is smaller by 128 Da when comparing the predominant hexa-acyl forms of each species through the use of shorter residues at most acyl positions. These differences in the acyl structure of psychrophilic lipid A in comparison to those of related mesophiles are constitutive analogs of those seen during the metabolic survival response of homeoviscous adaptation. They are likely to result in increased membrane fluidity, and suggest that evolutionary changes to constitutive lipid A structure were involved in adaptation to the cold-growth environments from which these psychrophiles were cultured. *P. cryohalolentis* also contains a strikingly high degree of acyl chain-length variability in single-carbon (-CH_2_-) increments. This same type of widely dispersed single-methylene variability has also been observed in the only other characterized psychrotolerant lipid A [31] suggesting that it may be a common feature of these organisms, which are capable of growth in a wide temperature range.
